# Response to long-term pharmacological management of metreleptin in a patient with monogenic obesity due to mutation in the LEP gene

**DOI:** 10.1186/s40842-024-00207-3

**Published:** 2025-07-07

**Authors:** Miguel Augusto O’Meara Novoa, Maria Camila Reyes Cajicá, Daniel Larrarte-Arenas, Jennifer Alexandra Palencia Ávila

**Affiliations:** 1 Department of Endocrinology, Compensar Salud: Compensar EPS, Bogotá, Colombia; 2 Pontifical Xavierian University, Bogotá, Colombia

**Keywords:** Leptin, LEP gene, Metreleptin, Congenital leptin deficiency, Novel mutation, Consanguinity, Obesity, Morbid

## Abstract

**Background:**

The cases of obesity worldwide have increased significantly, with this condition being considered a global pandemic. This pathology poses a major health problem due to its high morbidity burden. Its cause in the majority of cases is multifactorial; however, there are various cases of monogenic obesity reported in the literature. Mutation in the leptin gene causes a marked decrease in leptin levels, leading to intense hyperphagia and associated morbid obesity. Substituting leptin with metreleptin is a treatment option for these patients.

**Case presentation:**

We present the case of a patient with morbid obesity due to a single mutation in the LEP gene and approximately four years of treatment with metreleptin as a substitute therapy. Weight decreased from 154 to 64 kg and BMI decreased from 68.4 to 28.4 kg/m^2^.

**Conclusions:**

The patient achieved a reduction of 40 kg/m2 in BMI, corresponding to a body weight loss of 90 kg, with a significant improvement in associated metabolic comorbidities, acne, and hirsutism.

## Background

Obesity is a condition with significant morbidity due to its association with multiple metabolic disturbances in patients. There has been a documented increase in its prevalence, making it a global pandemic and a public health concern [1, 2]. It is largely a multifactorial disease with a substantial hereditary component [3]. Few cases of monogenic obesity exist, with the most frequently encountered mutation being in the melanocortin 4 receptor *(MC4R)*. Other related genetic mutations are found in leptin *(LEP)*, leptin receptor *(LEPR)*, proopiomelanocortin *(POMC)*, and prohormone convertase 1 *(PC1)* genes [4].

Leptin is a protein primarily secreted in a pulsatile manner by white adipose tissue and exerts its effects through binding to leptin receptors in the central nervous system, specifically in the hypothalamus. Elevated circulating levels are closely associated with increased body fat, reflecting energy reserve status. It encompasses various effects, including anorexigenic effects, energy homeostasis, neuroendocrine functions, and metabolic functions, among others [5].

Monogenic obesity results from homozygous mutations in the leptin-melanocortin system, leading to congenital leptin deficiency characterized by severe obesity with hyperphagia and multiple endocrine disorders such as diabetes mellitus, dyslipidemia, and ovarian dysfunction, among others [6]. This condition can be managed through exogenous administration of metreleptin, thereby substituting leptin levels to address the complications associated with total leptin deficiency as reported previously [7]. Given the rarity of this type of obesity, we present the case of a patient with a specific mutation in the *LEP* gene reported only once [8] and share our experience with treatment over recent years.

## Case presentation

We present a 31-year-old female patient with a history of morbid obesity since childhood, associated with amenorrhea. She was initially seen by Pediatric Endocrinology at the age of 9 years, with no significant maternal-perinatal history; birth weight and length were normal. However, over the years, the patient experienced progressive weight gain despite dietary management and exercise. At 16 years of age, she weighed 120 kg with a Body Mass Index (BMI) of 53 kg/m2, leading to the decision to undergo gastric sleeve surgery, resulting in transient weight loss of 20 kg (16.6%) with an end weight of 100 kg (BMI 44.4 kg/m2). Despite ongoing dietary management and physical activity, she gained approximately 41 kg over 5 years, weighing 141 kg (BMI 62.6 kg/m2).

Her family history includes consanguineous parents, a mother with grade I obesity, a paternal aunt with morbid obesity, and a younger sister with morbid obesity [8]. Due to the disease’s poor progression, in 2016, she underwent evaluation by Human Genetics, which recommended comprehensive genetic testing via DNA sequencing chromatogram. The report confirmed a homozygous mutation in the *LEP* gene variant *NM_000230:c.350G* > *T (NP_000221 1:p.Cys117Phe)*, indicating congenital leptin deficiency as the cause of monogenic obesity. This mutation was also identified in her younger sister, who exhibited a similar clinical presentation of morbid obesity since childhood.

**Fig. 1:**
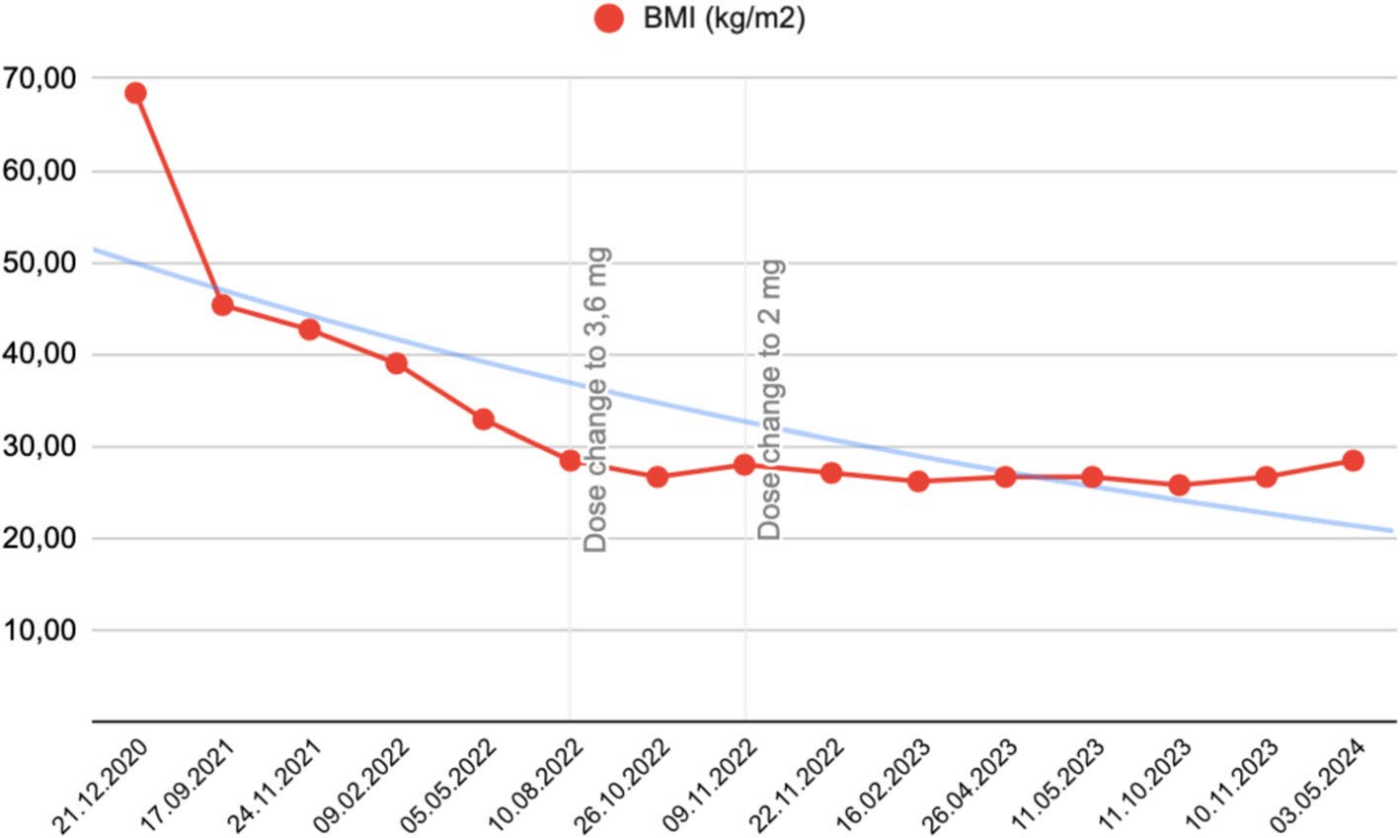
BMI reduction over time. Legend: BMI reduction over time (red line). Trendline of BMI reduction (blue line). Change in metreleptin dosage from 5 to 3.6 mg and to 2 mg (vertical gray lines)

Following the diagnosis of congenital leptin deficiency, monitoring of weight, BMI, metabolic parameters, among others, commenced. Additionally, in 2020, treatment with metreleptin replacement therapy was initiated when her weight was 154 kg (BMI 68.44 kg/m2) (Table 1 and Fig. 1).

**Table 1:** Weight, BMI for a height of 150 cm, metreleptin dose, DEXA

Date	Weight (kg)	BMI (kg/m2)	Metreleptin (mg SC daily)	DEXA (BMD)
21.12.2020	154	68,44	5,0	
17.09.2021	102	45,33		
24.11.2021	96	42,67		
09.02.2022	87,7	38,98		
05.05.2022	74	32,89		
10.08.2022	64	28,44	3.6	
26.10.2022	60	26,67		
09.11.2022	63	28,00	2,00	
22.11.2022	61	27,11		1280 g/cm2 (T Score 2.2σ)
16.02.2023	58,9	26,18		
26.04.2023	60	26,67		
11.05.2023	60	26,67		
11.10.2023	58	25,78		
10.11.2023	60	26,67		
03.05.2024	64	28,44		

*BMI* Body Mass Index, *SC* Subcutaneous, *DEXA* Dual-energy X-ray absorptiometry, *BMD* Bone mineral density (L1-L4)

Prior to commencing metreleptin, physical examination findings included severe obesity, acanthosis nigricans, mild hirsutism, acne on the chest region, and small hands and feet associated with clinodactyly and nail hypoplasia. During clinical follow-up, in addition to significant weight loss, the patient has shown improvement in acne and acanthosis nigricans, regular menstrual cycles, improvement in hepatic steatosis, gradual improvement in prediabetes, and obstructive sleep apnea syndrome (OSAS), currently without the need for continuous positive airway pressure (CPAP) machine. We do not have any laboratory tests prior to 2021. In the tests from 2021 to June 2023, there is evidence of gradual weight loss, improvement in prediabetes, and improvement in liver function tests. However, there is persistent mixed dyslipidemia due to low adherence to the nutritional plan, likely as a result of living in a rural area and irregular physical activity (Table 2).

**Table 2:** Laboratory results from 2021 to June 2023

Laboratory	2021	2022	2023	2023/06
Glucose (mg/dl)	86	87,7		
HbA1c (%)	5,40		5,70	5,50
Triglycerides (mg/dl)	76	70,3	72,5	58,4
Total Cholesterol (mg/dl)	145	206,9	210,6	212,2
HDL Cholesterol (mg/dl)	38	73	88,1	93
LDL Cholesterol (mg/dl)	95	119,8	108	107,5
Serum Creatinine (mg/dl)	0,56	0,71		0,72
Aspartate Aminotransferase (AST) U/L	48	15,5	26	26,3
Alanine Aminotransferase (ALT) U/L	27	9,8	37,8	28,2
Ultrasensitive TSH (uIU/ml)	2,01			
Free T4 (ng/dl)	0,98			
Total 25-Hydroxy Vitamin D (ng/mL)	18			23,4
Estradiol (pg/ml)	30,7			
FSH (mIU/ml)	5,11			
LH (mIU/ml)	1,88			
Serum Albumin (g/dl)	4,4			
17-Beta Estradiol (pg/ml)	23,5			
AM Cortisol (ug/dl)	6,19			

*TSH* Thyroid Stimulating Hormone, *FSH* Follicle Stimulating Hormone, *LH* Luteinizing Hormone

Despite the mutation diagnosis being made in 2016, metreleptin was initiated in 2020 following approval by the Colombian National Institute of Food and Drug Surveillance (INVIMA) [9]. Metreleptin administration began at 5 mg subcutaneously (SC) daily for an initial weight of 154 kg (BMI 68.44 kg/m2), resulting in a 58 kg (37.6%) weight loss over one year. At the beginning of treatment, the patient presented with headache, dizziness, and polydipsia, with these side effects later resolving. Due to weight stabilization not being achieved, the same dose was maintained until August 2022, when a stabilization in weight reduction and remission of obesity were observed. Consequently, the metreleptin dose was adjusted to 3.6 mg SC daily. Approximately one year later, weight and BMI curves remained stable, leading to another adjustment to a maintenance dose of 2 mg of metreleptin, which continues to date. Currently, the weight and BMI curves have remained stable, with a total weight loss of 90 kg and a reduction in BMI of 40 kg/m2 to date.

The patient is currently undergoing multidisciplinary follow-up, with associated comorbidities including dyslipidemia, hepatic steatosis and Polycystic Ovary Syndrome (PCOS) and subclinical hypothyroidism (Table 2).

## Discussion

Obesity has doubled its prevalence rate over the past 30 years, becoming a global pandemic [10]. This condition is multifactorial, where the interaction of the environment with various genetic components plays an important role [3]. Although the most common cause of obesity is considered multifactorial, there is a significant component of patients with monogenic obesity. The most frequently implicated genes include *MC4R*, *LEP*, *LEPR*, *POMC*, and *PC1*, among others, causing severe childhood obesity [4, 10].

Congenital leptin deficiency was first described in 1997 in two cousins with severe childhood obesity from a highly consanguineous Pakistani family, identifying a homozygous mutation in the *LEP* gene [11]. To date, several cases of different *LEP* gene mutations have been described, primarily in regions with high consanguinity rates. Due to the limited evidence in literature, it is challenging to establish a more accurate prevalence rate of monogenic obesity due to *LEP* gene deficiency. Some studies report an estimated prevalence rate of 3% among severely obese [12]. Therefore, the case presented represents a very rare cause of severe obesity. In our setting, in 2016, the patient in this case and her younger sister were identified with a unique mutation to date in the *LEP* gene variant *NM_000230:c.350G* > *T (NP_000221 1:p.Cys117Phe)* [8]. This condition is characterized by normal birth weight, associated with severe early-onset obesity. Additionally, it presents intense hyperphagia, suppression of blood leptin levels, hypothyroidism, hypogonadism, and hyperinsulinemia, among others [10, 13].

Metreleptin is an analog of recombinant human leptin that differs from human leptin by presenting a methionine residue at the N-terminal end of the polypeptide chain. Its primary effects are based on decreasing appetite and increasing the feeling of satiety, leading to weight loss. The impact of weight reduction through leptin replacement therapy has been widely studied, estimating a weight reduction rate of approximately 54% from the initial weight [14]. Additionally, it reduces elevated triglyceride levels and insulin resistance. At the reproductive level, this medication decreases androgen production and normalizes gonadotropin secretion [7]. This medication is administered once a day subcutaneously. The dose varies depending on the patient’s body weight. For patients weighing ≤ 40 kg, a dose of 0.06 mg/kg/day is administered, while for patients weighing > 40 kg, the daily dose is 2.5 mg and 5 mg for men and women, respectively [15]. Concomitantly, this medication has shown significant improvement in metabolic parameters such as triglycerides, total cholesterol, fasting glucose, and increased insulin sensitivity [16].

In approximately 4 years, the patient achieved a decrease of 40 kg/m2 in BMI, corresponding to 90 kg of body weight. This value corresponds to a weight reduction of approximately 60% from the initial weight, thus considerably exceeding the initial expectations based on scientific evidence [14]. Additionally, there was significant improvement in associated metabolic comorbidities, acne, and hirsutism.

Bariatric surgery as a weight reduction treatment is an alternative for these patients. A systematic review and meta-analysis of outcomes in patients undergoing bariatric surgery with follow-up beyond 10 years has shown a weight reduction of approximately 47% [17]. We consider the presented case highly relevant, as it identifies a patient with an underdiagnosed pathology, with a very rare mutation, who also shows an impact on weight reduction through metreleptin treatment that exceeds the expected for metreleptin and even greater compared to the results obtained in patients undergoing bariatric surgery [17]. Since bariatric surgery is a procedure that carries various risks, hospitalization, and costs, thus impacting the healthcare system, metreleptin is a treatment option to consider.

Given the multiple benefits of leptin replacement therapy with metreleptin mentioned above, the significant impact on weight reduction, even exceeding what is reported in the literature, leads to an important change in the natural history of this disease. Therefore, the importance of conducting a more active search for possible rare mutations in patients with severe obesity is emphasized.

## Conclusions

Monogenic obesity is one of the rare causes of obesity, with leptin deficiency due to *LEP* gene mutation being one of them. This condition involves early-onset severe obesity, associated with intense hyperphagia and various metabolic comorbidities. In this rare condition, metreleptin as a leptin replacement therapy has become one of the management options for these patients. Through this case, we have demonstrated a weight reduction impact greater than previously described in the literature. Our observations are highly useful for guiding the scientific community in the treatment of patients with leptin deficiency and secondary severe obesity. Additionally, they are helpful in guiding potential future scientific studies to determine possible benefits in the treatment of other endocrine and metabolic diseases. We emphasize that the result in weight reduction, improvement in metabolic parameters, and therefore the impact on the natural course of the disease highlights the importance of actively searching for possible rare causes of obesity.

## Data Availability

Not applicable.

## Abbreviations

MC4R: Melanocortin 4 receptor
LEP: Leptin
LEPR: Leptin receptor
POMC: Proopiomelanocortin
PC1: Prohormone convertase 1
BMI: Body Mass Index
INVIMA: Instituto Nacional de Vigilancia de Medicamentos y Alimentos (Colombian National Institute of Food and Drug Surveillance)
SC: Subcutaneously
PCOS: Polycystic Ovary Syndrome
OSAHS: Obstructive Sleep Apnea Hypopnea Syndrome
CPAP: Continuous Positive Airway Pressure
